# Dietary practice and associated factors among type 2 diabetic patients attending chronic follow-up in public hospitals, central Ethiopia, 2022

**DOI:** 10.1186/s12913-023-10293-1

**Published:** 2023-11-17

**Authors:** Dureti Tirfessa, Mitsiwat Abebe, Jiregna Darega, Mecha Aboma

**Affiliations:** Department of Public Health, Ambo University College of Medicine and Health Sciences, P.O Box 19, Ambo, Ethiopia

**Keywords:** Type 2 diabetes, Dietary practice, West Shewa Zone Public Hospital

## Abstract

**Background:**

Diabetes Mellitus (DM) is affecting numerous Ethiopian populations regardless of environmental and social status. Diabetic people all over the world are commonly urged to acquire a healthy eating habit, which necessitates lifelong changes in food habits, beliefs, and meal patterns. Dietary management is considered one of the cornerstones of diabetes care, as it is an important component of the overall treatment plan. Choosing and following a healthy diet is important for everyone, especially people with diabetes.

**Objective:**

This study aims to assess dietary practices and associated factors among type 2 diabetes patients in the west Shewa Zone, Oromia Regional State, Ethiopia, in 2022.

**Methods:**

A hospital-based cross-sectional study design was conducted in West Shewa Zone public hospitals among 421 randomly selected type 2 diabetic patients from February 1 to March 30, 2022. Data were collected using a structured and pre-tested interviewer-administered questionnaire. Descriptive, bivariate, and multivariate binary logistic regression analyses were done using SPSS.

**Results:**

In this study, about 35.6% (95% CI: 30.9–39.9) of type 2 diabetes patients had good dietary practices. Diabetes knowledge (AOR 9 2; 95% CI 4.4–19.4), food-secured households (AOR 3.3; 95% CI 1.6–6.9), high self-efficacy (AOR 6.6; 95% CI 3.2–13.9), diabetes diet information from healthcare professionals (AOR 2.9; 95% CI 1.3–6.4), complete dietary change (AOR = 2.3; 95% CI 1.1–4.8), and female gender (AOR 3.6; 95% CI 1.6–8.1) were independent predictors of good dietary practice.

**Conclusion:**

The proportion of patients with type 2 diabetes, who attended follow-up at West Shawa Public Hospitals and practiced good dietary habits, was low. Patients' household food insecurity, diabetes knowledge, self-efficacy, source of information on the diabetic diet, complete dietary change after diabetes diagnosis, and gender were all significantly associated with type 2 diabetic patients' dietary practices. Thus, promoting the provision of continuous, modified, and comprehensive education and advice on the importance of diabetes self-management, particularly adherence to dietary recommendations, is fundamental to decreasing the burden of diabetes complications and massive health expenses among diabetic patients.

## Introduction

Diabetes mellitus (DM) is a group of physiological abnormalities characterized by hyperglycemia caused by insulin resistance, insufficient insulin production, or overproduction of glucagon [1]. Between 2000 and 2019, the number of deaths from diabetes grew globally by 70% with an 80% increase in deaths among males. In countries with low- middle income, the number of deaths from this disease has nearly doubled since 2000. Diabetes is the 6th leading cause of death among upper- middle income countries, and there is evidence that it is epidemic in many developing and newly industrialized nations [2, 3]. The reasons for this growth are numerous, and include a variety of factors as a result of sedentary living, high-energy dietary intakes, and other factors that are still unknown [4]. Diabetes complications such as coronary artery and peripheral vascular disease, stroke, diabetic neuropathy, amputations, kidney failure, and blindness are increasing disability, decreasing life expectancy, and causing massive health-care costs in almost every country [3].

A healthy diet practice is among of the seven self-care practices recommended by the American Association of Diabetes Educators for effective diabetes management [5]. Diabetes patients must exercise strict control over their lives, which necessitates psychological and behavioral changes, as well as self-management [6, 7]. A balanced lifestyle that includes regular physical activity and good nutrition is essential for people with type 2 diabetes to achieve and maintain appropriate glycemic control [8]. It is critical to engage in diabetes-related self-care practices in order to reduce the disease's complications [9].

The process of actively engaging in self-care activities with the goal of improving one’s behavior and well-being is known as self-management [10]. Many patients find it extremely difficult to implement long-term self-care standards and accept life limitations, adding to the psychological pressures they face. These people require special attention when it comes to diabetes self-management, including physical, psychological, and social support [11]. Addressing all aspects of self-care management for type 2 diabetes patients, including blood glucose monitoring, nutrition, exercise, medication, and foot care, is a significant challenge for health-care practitioners and the health-care system [12].

Diet is an essential component of self-management in any situation [13]. People with type 2 diabetes, in particular, are advised to consume whole grains, beans, fruits, and non-starchy vegetables, which provide fiber as well as important vitamins and minerals [14–16]. It has two functions in diabetes mellitus. The first is to provide diabetic patients with appropriate nutrition so that they can live their lives as healthy people do, and the second is to provide nutrients in a balanced form so that they can maintain a healthy body [17]. Failure to do so increases the risk of diabetic complications developing early and causing micro and macro vascular complications [7].

Overall, type 2 DM risk factors that contribute to disability and death could be metabolic, such as a high BMI, or behavioral, such as an inappropriate diet, smoking, and a sedentary lifestyle with little physical activity [18]. A new and comprehensive therapeutic approach can be provided by lifestyle promotion. It is capable of providing patients with highly effective tools for disease control and quality of life improvement [19].

Dietary control is an essential component of all type 2 diabetes treatment and the safest method of control; one-third of patients can maintain a satisfactory blood glucose level solely through diet control [12]. More than half of Ethiopian diabetes patients do not follow the dietary recommendations of their doctors and nutritionists [20, 21]. The proportion of patients who were successful in controlling their fasting blood sugar remained low (30.2%). Their diets were deficient in energy and were unbalanced [22]. In terms of choosing a balanced diet, meal planning, calculating food calories, and regulating dietary behavior, the majority of patients with poor glycemic control did not adhere to a diabetic meal plan [23].

Despite being aware of their obligation to self-manage their diabetes, some patients found it difficult to apply what they had learned in diabetes classes and to find the personal motivation and resources required to make the significant behavioral changes required [24]. Patients consistently emphasized the causal links between good eating, stressful life events related to money, the health-care system, and discrimination, and stress related to diabetes, its stigma, and management [25].

Diabetes patients go to the hospital every three months. During their scheduled visits, they will receive more checks on their prescriptions and drug adherence, but less on their dietary practices. In addition, caregivers pay less attention to the patients’ dietary recommendations. This could explain why many diabetic patients' blood glucose levels remain elevated after treatment, raising the risk of disease progression and complications. The benefit of lifestyle management is obvious; however, better paradigms for targeting, individualizing, and maintaining the effects are required [26]. To that end, data on dietary practices among type 2 diabetes mellitus patients in Ethiopia are limited.

**Fig. 1:**
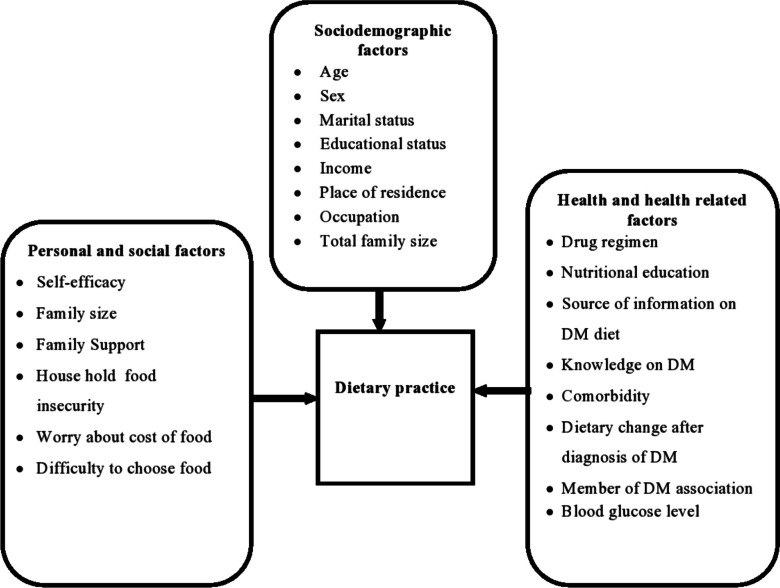
Conceptual frame work to assess the dietary practice and its associated factors among type 2 diabetic patients in West Shewa Zone, Oromia Regional State, Ethiopia, 2022 [27–30]

Until now, little evidence has been available on dietary practices, mainly focusing on factors associated with dietary practices among type 2 DM patients. This was not well explored, particularly in the cases of central Ethiopia, specifically patients receiving chronic care in West Shewa. Hence, this study will contribute to filling this knowledge gap, highlight the magnitude of the problematic dietary practices among patients, and identify the facilitators and barriers to adhering to dietary recommendations. Thus, this study aimed to assess the dietary practices and associated factors among type 2 diabetic patients in West Shawa public hospitals (Fig. 1).

## Methods

### Study area and period

A hospital-based cross-sectional study design was conducted in public hospitals in the West Shewa zone, Oromia regional state, central Ethiopia from April 1 to June 30, 2022. West Shewa zone has 22 districts, and the districts are sub-classified into urban and rural kebeles. In Ethiopia, a kebele is the smallest administrative unit. West Shewa zone has nine public hospitals, namely; Incinni primary hospital, Ginchi primary hospital, Jeldu primary hospital, Gindeberet general hospital, Ambo general hospital, Ambo referral hospital, Guder primary hospital, Gedo general hospital, and Bako primary hospital [31].

### Study design, sample size, and sampling procedures

A hospital-based cross-sectional study design was used among type 2 diabetes patients receiving chronic care at public hospitals in the west Shewa Zone of Ethiopia’s Oromia Regional State. This study included all type 2 diabetic patients who had been on regular follow-up for at least 6 months and were over the age of 18. The study excluded type 2 DM patients who were psychotic or critically ill, incomplete data, as well as pregnant and breast-feeding women. The sample size was calculated using the single population proportion formula with the assumption of the proportion of type 2 diabetic patients with good dietary practices (48.6%), a 95% confidence level, a 5% margin of error, and a 10% non-response rate. The largest sample size, 421, was generated [30].

**Fig. 2:**
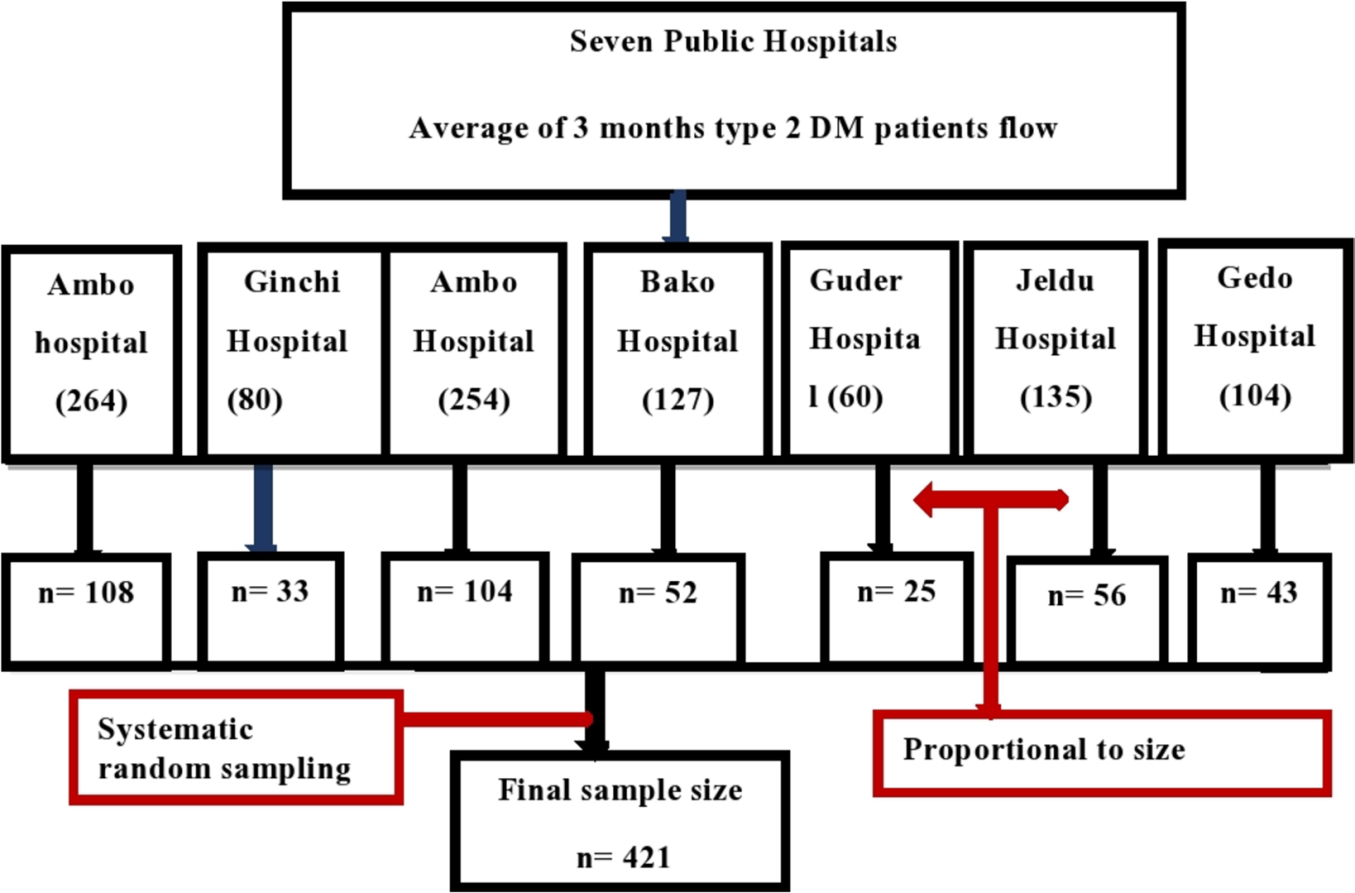
Schematic presentation of sampling procedures for type 2 diabetic patients in attending follow-up at a public hospital in West Shewa Zone, Oromia Regional State, Ethiopia, February to March 2022

Seven of the nine public hospitals in the West Shawa Zone that provide chronic follow-up services were included in this study, while two were difficult to reach due to security concerns during data collection and were therefore excluded. Each hospital provided a three-month average of the number of type 2 DM patients attending chronic follow-up prior to the data collection period. Based on this, the study’s calculated sample size was allocated to each hospital in proportion to the number of type 2 DM patients who attended their chronic follow-up clinics. The study unit (type 2 diabetic patients attending chronic follow-up) in each hospital was then selected at two-patient intervals using systematic random sampling techniques (Fig. 2).

### Data collection tools and personnel

Data was collected by seven BSc nurses under the supervision of three health officers using a pretested, structured, interview-administered questionnaire adapted and modified from similar literature [30–37]. The questionnaire was translated into the local languages and retranslated to English to assure its consistency by different language experts. The questionnaire consists of sociodemographic characteristics, health and health-related factors, and personal and social factors for type 2 diabetic patients. Primary data were gathered from study participants, while clinical data such as co-morbidity history, diabetes duration, and blood glucose level were extracted from patients’ medical records using a structured, guided checklist. Cronbach’s alpha was checked for self-efficacy, family support and house hold food security status with the value of 0.88, 0.99 and 0.81, respectively.

### Terms and operational definitions

#### Dietary practice

Dietary practice of over the past two weeks was assessed using the modified form of the fourteen item scales taken from related literature [30, 32]. The items focus on the short- and long-term dietary plans of patients, their attitude towards preparing a diabetes diet, the selection of food items in their daily meal, and the pattern of food intake within a day. The items had a “Yes” or “No” response. Value 1 was given for the “yes” response and 0 for the “no” response. Those who scored below the mean value were categorized as having poor dietary practices, while those who scored equal to or above the mean value were classified as having good dietary practices.

#### Self-efficacy

The ability of type 2 diabetes patients to sustain their eating practices was assessed using a self-efficacy questionnaire. Self-efficacy was examined in this study using 9 items of modified self-efficacy for diet from 15 items of the diabetes management scale [33], with 0 being the lowest scale and 9 representing the highest scale. Components were calculated by using the mean value to categorize the respondents, as the total score ranges from 0 to 9, with a higher score indicating a higher level of self-efficacy. In this study, self-efficacy scores were classified into two levels: high self-efficacy (6-9) and low self-efficacy (0–5), while 1 was given for “yes” and 0 was given for “no”.

### Knowledge on diabetes

The knowledge of the patients about diabetes was measured by using nine variables with 24 possible correct responses adopted from similar literature. Patients’ knowledge about diabetes was similarly calculated by taking the mean values of the questions and labeling them as having good or poor knowledge for values above and below the mean value, respectively [34].

### Family support

The family support questionnaire was used to assess the family’s assessment of the support system and motivation to assist type 2 diabetes patients in adopting healthy eating habits. The Diabetic Social Support Questionnaire-Family (DSSQ-Family), developed by La Greca and Bearman, will be used to assess family support (cited in Puntsho Om) [35]. “Never”, “less than 2 times a month”, “twice a month”, “once a week”, “many times a week”, and “at least once a day” were used on the DSSQ-frequency family’s rating scale. The DSSQ-Family total score ranges from 0 to 100 and is classified as low social support (0–33), moderate social support (34–66), and high social support (67–100).

#### Household food insecurity

Questions about household food insecurity were measured using the Food and Nutrition Technical Assistance (FANTA) tool called the Household Food Insecurity Access Scale (HFIAS) [36]. The respondents were asked about the occurrence of the condition, that is, whether the condition in the question happened at all in the past four weeks (yes or no). For respondents who answered “yes” to the occurrence question, the frequency of occurrence of the condition will be asked to determine whether the condition rarely happened (once or twice), sometimes (three to ten times), or often (more than ten times) in the past four weeks.

Food security status was computed using the HFIAS occurrence and frequency questions, and the Insecurity Access Scale score was analyzed based on the HFIAS criteria and categorized into food-secure and food-insecure households.

#### Blood glucose level

For diabetic patients, blood glucose levels are controlled if FBG is less than 130 mg/dl and poorly controlled if it is greater than 130 mg/dl [37].

### Data management and analysis

Data quality was assured through pre-testing the data collection tools on 5% of the total sample size before they were used for actual data collection in a similar population that was not included in the study subjects. The principal investigator trained data collectors and supervisors for one day on the study instruments and consent form how to conduct interviews, and data collection procedures. Before data entry, the questionnaires were checked for completeness and consistency, and correction measures were taken by supervisors and investigators.

Following that, the data were coded, entered into EPI-Data version 4.6, and exported to SPSS software version 25 for data processing, cleaning, and analysis. To describe sociodemographic characteristics, health and health-related factors, personal and social factors, and dietary practices of type 2 diabetic patients, descriptive analysis such as frequency and percentage, mean, and standard division were used, and the results were presented in texts, tables, and graphs.

To identify factors associated with type 2 diabetic patients’ dietary practices, bivariable and multivariable analyses were performed using a binary logistic regression analysis model. The bivariable logistic regression model was used to identify candidate variables for the final model (multivariable logistic regression) at a *p*-value less than 0.25, and the final model, multivariable logistic regression, was used to see the independent effect of each explanatory variable on the study variable after adjusting for other variables that entered the multivariable model. An adjusted odds ratio with a 95% confidence interval and a *p*-value of 0.05 was reported to have a significant association with type 2 diabetes patients’ dietary practices.

To assess model fitness, the Hosmer-Lemshow goodness-of-fit (*P*-value = 0.348) was calculated. The independent variables were tested for multicollinearity using the Variance Inflation Factor (VIF) and Tolerance Tests, and no variables with a VIF greater than 2 were excluded from the analysis.

## Results

### Sociodemographic characteristics of study participants

A total of 421 respondents participated in this study, making the response rate 100%. Of the study participants, 284 (67.5%) were male. The mean ages of the respondents were 59.13 (± 11.58 SD) with a range of 28–83 years. More than half of the respondents, 233 (55.3%), were over the age of 60. The majority of respondents, 350 (83.1%), were married, and 282 (67%) of them were urban residents.

Almost half, 215 (51.1) of the study participants, had a total of 5 or fewer family members. Nearly one third (31.4%) of respondents attended college or higher education, and 116 (27.5%) of participants were self-employed. The majority of study participants 382 (90.7%) were worried about the high cost of food (Table 1).

**Table 1:** Sociodemographic characteristics of type 2 diabetic patients attending follow-up at a public hospital in West Shewa Zone, Oromia Regional State, Ethiopia, February to March 2022

**Variable** (*n* = 421)	**Category**	**Frequency**	
		**Number**	**Percent (%)**
Sex	Female	137	32.5
	Male	284	67.5
Age	18–59	188	44.7
	≥ 60	233	55.3
Residence	Urban	282	67.0
	Rural	139	33.0
Marital status	Single	10	2.4
	Married	350	83.1
	Divorced	25	5.9
	Widowed	36	8.6
Ethnicity	Oromo	384	91.2
	Amhara	32	7.6
	*Others	5	1.2
Religion	Orthodox	264	62.7
	Protestant	131	31.1
	Muslim	21	5.0
	**Others	5	1.2
Educational status	Unable to read and write	111	26.4
	Primary school (1–8)	99	23.5
	Secondary school (9–12)	79	18.8
	College& above	132	31.4
Occupation	Farmer	92	21.9
	Daily laborer	54	12.8
	Government employee	107	25.4
	Self-employee	116	27.6
	Housewife	18	4.3
	***Others	34	8.0
Total family size	≤ 5	215	51.1
	> 5	206	48.9
Monthly income	Low	27	6.4
	Moderate	180	42.8
	High	214	50.8
Worry about high cost of food	Yes	382	90.7
	No	39	9.3

^*^Others (Ethnicity): Tigre, Gurage, and Wolayita, ^***^Others (occupation): NGO, Student, ^**^Others (Religion): Catholic, Wakefata, and indicated in table

### Health and health-related characteristics of study participants

The duration of the disease for 224 (53.2%) of the studied participants was ≤ 6 years. Two thirds, or 256 (60.8%) of the study participants, had co-morbidities, mainly hypertension (39.4%), kidney disease (7.6%), and heart disease (6.9%). Of the total, only 165 (39.2%) of the study participants received nutritional education on the diabetes diet at the hospital during their regular follow up. One hundred seventy-nine (42.5%) of the study participants made a complete change in their dietary habits when they knew they were diabetic.

Regarding medication regimens, 361 (85.7%) of the study participants were on oral medication, while 59 (14%) of them were on insulin injection. Of the study participants, only 33 (8.8%) were members of the diabetes member association. The sources of information on the diabetes diet for 152 (36.1%) and 122 (29%) of the studied participants were health professionals and diabetic patients, respectively. One hundred sixty-three (38.7%) of study participants had controlled (FBG ≤ 130) blood glucose levels (Table 2).

**Table 2:** Health and health-related characteristics of type 2 diabetic patients attending follow-up at a public hospital in West Shewa Zone, Oromia Regional State, Ethiopia, February to March 2022

Variable	Category	Frequency	
		**Number**	**Percent (%)**
Duration of DM (*n* = 421)	< = 6	224	53.2
	> 6	197	46.8
Drug regimen	Insulin injection	59	14
	Tablets	361	85.7
	Diet	1	0.2
Presence of comorbidity	Yes	256	60.8
	No	165	39.2
Type of comorbidity	Hypertension	166	39.4
	Heart disease	29	6.9
	Nerve disease	7	1.7
	Kidney disease	32	7.6
	Eye disease	22	5.2
Nutrition education given in Hospital	Yes	165	39.2
	No	256	60.8
Dietary changed after diagnosis	Yes	179	42.5
	No	242	57.5
Source of information on diabetes diet	Health professionals	152	36.1
	Diabetes patients	122	29
	*Others	147	34.9
Member of DM association	Yes	37	8.8
	No	384	91.2
Blood glucose level	≤ 130 mg/dl	163	38.7
	> 130 mg/dl	258	61.3

^*^Others (source of information): TV/Radio, neighbors, social media, and indicated in the table

### Knowledge about diabetes mellitus and dietary practices of study participants

**Fig. 3:**
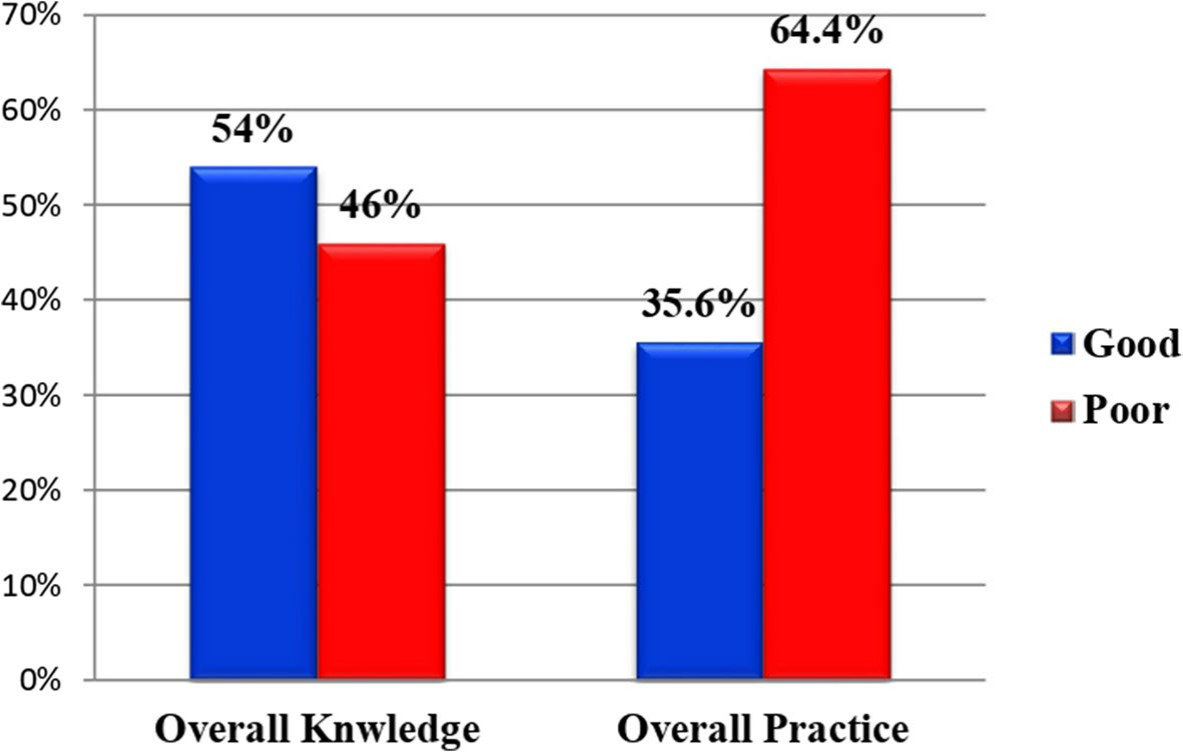
Knowledge and dietary practice among type 2 diabetic patients on follow-up at a public hospital in West Shewa Zone, Oromia Regional State, Ethiopia, February to March 2022 (*n* = 421)

Of the total study participants, 194 (46.1%) had good knowledge regarding diabetes mellitus, whereas 227 (53.9%) had poor knowledge. Overall in this study, about 150 (35.6%) of study participants had good dietary practice while 271 (64.4%) of study participants had poor dietary practice (Fig. 3).

### Personal and social factors of study participants

**Fig. 4:**
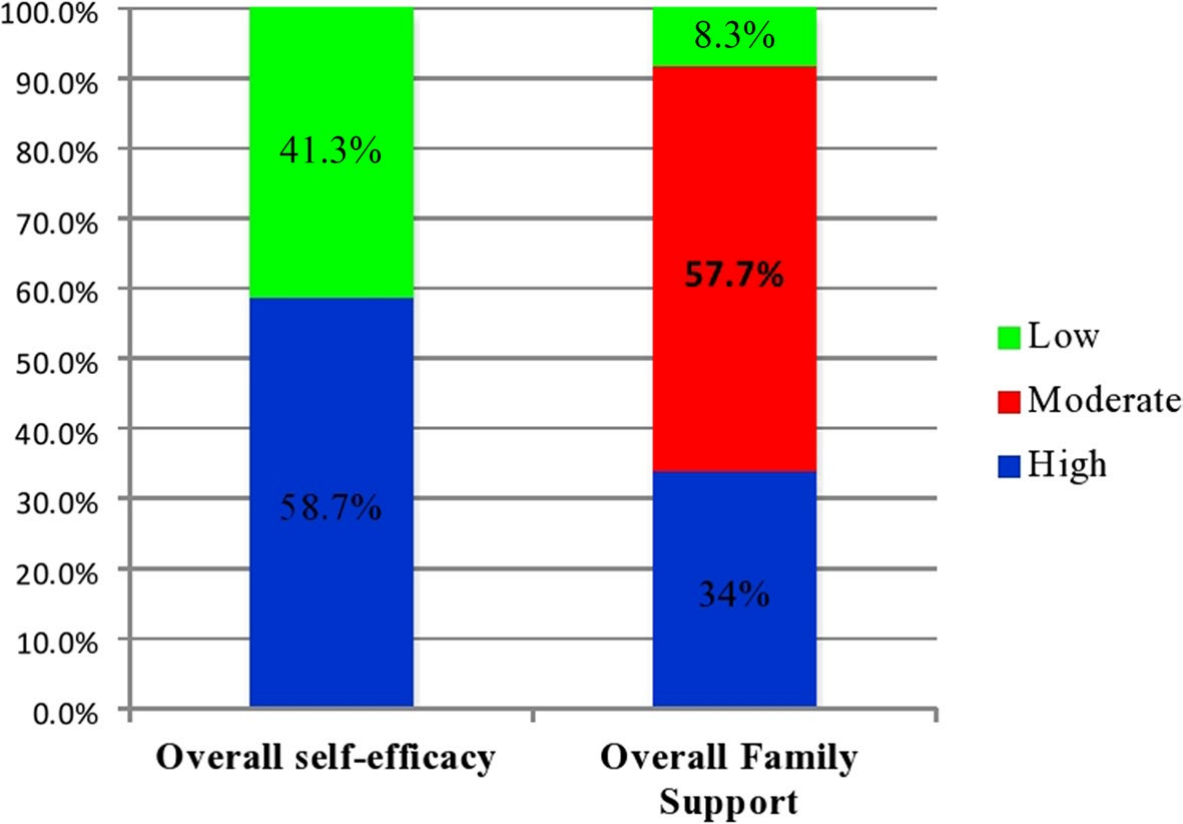
Self-efficacy and family support of type 2 diabetic patients attending follow-up at a public hospital in West Shewa Zone, Oromia Regional State, Ethiopia, February to March 2022

Overall, about 174 (41.3%) of the study participants had high self-efficacy, whereas 247 (58.7%) of them had low self-efficacy. Similarly, 143 (34.0%) of study participants reported high family support, 243 (57.7%) reported moderate family support, and 35 (8.3%) reported low family support (Fig. 4).

**Fig. 5:**
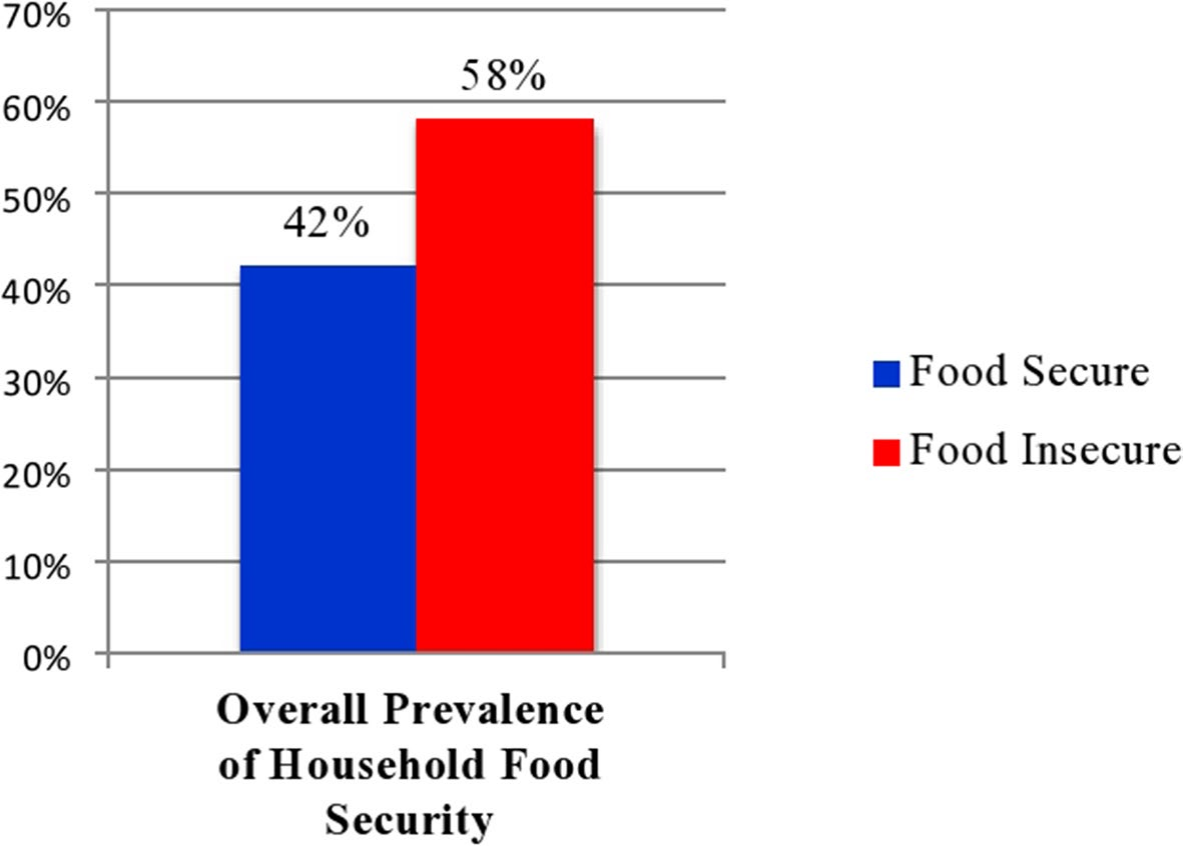
Household food security of type 2 diabetic patients attending follow-up at a public hospital in West Shewa Zone, Oromia Regional State, Ethiopia, February to March 2022

In this study, 177 (42%) and 244 (58%) of the study participants live in food-secure and food-insecure households, respectively (Fig. 5).

### Factors related to dietary practice

Multivariate binary logistic regression analysis revealed that patient knowledge, self-efficacy, food security, source of information, changing dietary habits, and sex were significantly associated with dietary practice after controlling for potential confounders. As a result, study participants with good diabetes mellitus knowledge were 9.2 times more likely to have good dietary practices than those with poor knowledge (AOR 9.2; 95% CI 4.4–19.4). When compared to those with low self-efficacy, those with high self-efficacy were 6.6 times more likely to have good dietary practices (AOR 6.6; 95% CI 3.2–13.9). Study participants from food-secure households were 3.3 times more likely to have good dietary practices compared to study participants from food-insecure households (AOR 3.3; 95% CI 1.6–6.9).

When compared with study participants who get information on diabetes diet from other sources such as TV, radio, and their neighbours, those who get information from health professionals were 2.9 times more likely to have good dietary practices (AOR 2.9; 95% CI 1.3–6.4). When compared to their counterparts, study participants who had made a complete change in their dietary habits after knowing they were diabetic were 2.3 times more likely to have good dietary practices (AOR 2.3; 95% CI 1.1–4.8). The odds of having good dietary practices among female study participants were 3.6 times higher compared to their counterparts (AOR 3.6; 95% CI 1.6–8.1) (Table 3).

**Table 3:** A bi-variable and multivariable logistic regression model showing factors associated with the dietary practice of patients with type 2 diabetes attending follow-up at a public hospital in West Shewa Zone, Oromia Regional State, Ethiopia, February to March 2022

Variable (*n* = 421)	Category	Dietary practice		COR (95% CI)	AOR (95% CI)	*P*-value
		Good	Poor			
Knowledge on DM	Poor	18	209	1	1	1
	Good	132	62	24.7 (14.0,43.6)	9.2 (4.4,19.4)	< 0.001*
Self-efficacy	Low	21	226	1	1	1
	High	129	45	30.9 (17.6,54.1)	6.6 (3.2,13.9)	< 0.001*
Source of information	Health Professional	102	50	5.5 (3.3,8.9)	2.9 (1.3,6.4)	0.011*
	DM patient	8	114	0.3 (0.1,0.4)	0.8 (0.3,2.2)	0.59
	Others	40	107	1	1	
Total family size	≤ 5	119	96	6.9 (4.4,11.16)	2.1 (0.9,4.6)	0.055
	> 5	31	175	1	1	
Marital status	Married	135	215	2.34 (1.3,4.3)	2.3 (0.9,6.0)	0.09
	Others	15	56	1	1	
Dietary change	Yes	110	69	8.1 (5.12,12.67)	2.3 (1.1,4.8)	0.023*
	No	40	202	1	1	1
Sex	Female	82	55	4.7 ( 3.1,7.3)	3.6 (1.6,8.1)	0.002*
	Male	68	216	1	1	1
Household food security	Food Secure	109	68	7.9 (5.1,12.5)	3.3 (1.6,6.9)	0.001*
	Food insecure	41	203	1	1	1
Member of DM association	Yes	20	17	2.3 (1.2,4.5)	2.6 (0.79,8.3)	0.11
	No	130	254	1	1	
Blood Glucose	Controlled	94	69	4.9 (3.2,7.6)	1.7 (0.8,3.6)	0.19
	Poorly controlled	56	202	1	1	
Education	Primary and less	36	174	1	1	
	Secondary and above	114	97	5.7 ( 3.6, 8.9)	0.3 (0.3,1.6)	0.37
Residence	Urban	131	151	5.48 (3.2,9.38)	1.9 (0.6,6.1)	1.9
	Rural	19	120	1	1	
Age	18–59	99	89	3.9 ( 2.6, 6.1)	1.6(0.7,3.5)	0.28
	≥ 60	51	182	1	1	
Family support	Low	9	26	1	1	
	Moderate	42	201	0.69 (0.3,1.6)	1.2 (0.3, 5.2)	0.86
	High	99	44	7.32 (3.1,17.3)	2.1 (0.4,9.9)	0.35
Worry about cost of food	Yes	143	239	2.74 (1.2,6.4)	1.8 (0.4,8.8)	0.48
	No	7	32	1	1	
Duration of DM	≤ 6	125	99	8.7 ( 5.3, 14.3)	0.7 (0.3,1.8)	0.47
	> 6	25	172	1	1	
Nutrition education at Hospital	Yes	105	60	8.2 (5.22,12.9)	1.3 (0.6,3.5)	0.48
	No	45	211	1	1	
Comorbidity	Yes	53	203	1	1	
	No	97	68	5.5 (3.54, 8.4)	1.2 (0.4, 2.9)	0.68

^*^Significant at *p* < 0.05, *AOR* Adjusted Odds Ratio, *CI* confidence Interval,1 = reference

## Discussion

The result of this study revealed a low proportion of good dietary practice among study participants, which contributes to raising the likelihood of diabetic problems developing early and developing micro- and macro-vascular complications. The findings of this study revealed that the overall magnitude of good dietary practice among type 2 diabetics was 35.6% (95% CI 30.9, 39.9). This result was consistent with the studies conducted in Bahir Dar and Jimma, Ethiopia, which revealed that the magnitude of good dietary practices among type 2 diabetics was 35.9% and 36%, respectively [27, 28].

However, the result of this study was lower than the studies conducted in other parts of Ethiopia: Gonder (46.7%), Addis Ababa (48.6%), Hawassa (55.8%), and Nigeria (76%), which revealed a high proportion of good dietary practices among diabetic patients [30, 34, 38, 39]. These could also be due to differences in the study settings, sample size, study design, and economic domains of study participants, as well as the type of tools used to measure these outcomes.

In contrast, we found a higher proportion of good dietary practices among type 2 diabetic patients than in studies conducted in Tikur Anbessa Specialized Hospital, Addis Ababa, Ethiopia (22.2%), and Hodeida City, Yemen (21% [40, 41]).

Differences in respondents' sociodemographic status, the time gap between studies, economic conditions, physical and financial accessibility, disease pattern, and health service issues; level of awareness; access to information, such as through mass media and other social media; family and peer relationships; cultural beliefs; as well as differences in study design, study area, study period, study population, and sample size, all contribute to the variation. Similarly, nowadays, information about diabetes nutrition is easily and quickly available at nearby private and public hospitals, and improvements in technology have made patients have a better understanding of the diabetic diet.

The result of this study showed that type 2 diabetic patients who had good knowledge of diabetes were 9.2 times more likely to have good dietary practices as compared to diabetic patients who had poor knowledge. This study finding was consistent with the study findings reported from Arba Minch and Bale, Ethiopia, which revealed that knowledge has a significant effect on self-care behaviors related to diet [42, 43]. The possible explanation for this might be that as the knowledge of diabetic patients about diabetes increases—especially how to prevent complications, how bad it is if complications occur, how to control blood glucose levels, and the importance of following good dietary practices—they will be more motivated and continue to maintain good dietary practices. Thus, good dietary practice is a pillar of diabetes self-management. In general, poor dietary practices are associated with low diabetes knowledge among diabetic patients, and good dietary practices improve as knowledge levels rise [44].

The findings of this study indicated that diabetic patients who lived in food-secure households were 3.3 times more likely to have good dietary practices than patients who lived in food-insecure households. This finding is consistent with a previous study, which showed that diabetic patients who resided in food-secure households had good dietary practices compared to those who lived in food-insecure households [42, 45]. It would be challenging for patients to practice dietary recommendations while there was an inadequate food supply within the household. As a result, diabetic patients who live in food-insecure households may use unhealthy coping mechanisms, such as reducing the frequency and amount of meals; less expensive and calorie-dense food consumption may also play a significant role in having poor dietary practices [46–48].

This study's findings revealed that self-efficacy was a significant predictor of good dietary practice. Thus, diabetic patients who had high self-efficacy were 6.6 more likely to have good dietary practices as compared to their counterparts. This finding was consistent with the study findings from Hungary, China, and India, which showed a significant positive relationship between good dietary practice and self-efficacy [49–51]. This expression can be explained by the fact that therapy for patients with diabetes requires lifestyle changes but faces difficulties at the beginning; to overcome these difficulties, they need to convince themselves, practice, and have high self-efficacy. Diabetic patients with low self-efficacy were stopped by obstacles and difficult situations and began to focus on the negative consequences of the disease rather than following recommended self-care practices [50, 52]. Thus, individual dietary choices are influenced by personal priorities, confidence, and self-determination.

This study found that patients who received information on diabetes diet from healthcare professionals were 2.9 times more likely to have good dietary practices as compared to those who received information from other sources.

The result of this study was consistent with the study findings from southwest Ethiopia and Bahrain, which showed that patients who had received healthcare professionals’ instructions or advice regarding diet had good dietary practices compared to those who had not received dietary advice from healthcare professionals [27, 53]. This evidence might be explained by the fact that the information disseminated by healthcare professionals was appropriate, trustworthy, and scientific, which is vital and the first step to making healthy dietary choices.

A large proportion of diabetic patients in this study, 60.8%, did not receive nutritional education on diabetes diets at the hospital, which may have prevented them from receiving diabetes diet information from healthcare professionals. This could have an impact on the healthy dietary practices of patients in the study area.

The finding of this study showed that, good dietary practice was 2.3 times more likely higher among diabetic patients who made complete dietary changes after diagnosis of DM compared to those who did not change their dietary habits. Thus, patients who made complete dietary habit change after diagnosis of DM had good dietary practice. Similarly, previous study findings from Athens, Greece and United Arab Emirates revealed improvement in eating habit might increase adherence to dietary recommendations among diabetic patients [54, 55]. Early changing dietary habit might help patients to reap the benefits of good nutrition earlier and insure their health; this can be great motivation for them to maintain good dietary practice.

The findings of this study indicated that good dietary practice was 3.6 times more likely among female diabetic patients as compared to their counterparts. This finding was consistent with previous research from north and south-western Ethiopia, rural south India, and Yemen [27, 41, 56]. The possible explanation for this might be that women were more amenable to changing their diet than men. Furthermore, because cooking is primarily done by women in most Ethiopian societies, this allows them to prepare foods that are in accordance with their diet plan. However, the current study findings contradict a Nepalese study that found females were less likely than men to adhere to recommended dietary practices [44]. This disparity could be due to socioeconomic and cultural differences, as well as the study setting.

### Limitation of the study

The limitation of the study is that dietary practices were evaluated using self-reported data rather than direct observation. Additionally, recall and social desirability bias might have been present in the tool used to obtain data about household food insecurity and family support.

## Conclusion

The proportion of good dietary practices among patients with type 2 diabetes attending follow-up at a public hospital in West Shewa Zone was found to be lower than that in many African countries, as well as in other parts of Ethiopia. Diabetic patients’ household food security status, knowledge about diabetes mellitus, self-efficacy, source of information on the diabetic diet, dietary habit change after diagnosis of DM, and sex have statistically significant associations with the dietary practices of type 2 DM patients.

The Zonal Health Bureau should develop health information dissemination programs and strategies to improve diabetic patients’ knowledge of diabetes and the importance of diet-based diabetes management. A routine health information dissemination plan should be developed, and comprehensive education about diabetes and diabetes self-management, particularly dietary recommendations, should be promoted by hospitals. Healthcare professionals should regularly provide diabetic nutritional information, and education should focus on increasing patients’ diabetes knowledge. The importance of maintaining good dietary practices and their impact on the reduction of diabetes-related complications, blood glucose control, and a healthy lifestyle should be emphasized. Understanding these improves self-efficacy, which leads to better dietary practice.

Type 2 DM patients bear full responsibility for increasing their diabetes knowledge, self-efficacy, and food security in order to adhere to the recommended dietary practices. They should also seek nutrition advice from health professionals. Participation of the media and non-governmental organizations in improving knowledge and type 2 diabetes patients’ dietary practices is highly recommended, as diabetic care necessitates collaborative efforts.

The evidence obtained from this study will also contribute to improve dietary self-care support by health professionals and caregivers, gives guide for policy makers with updated information for future planning and interventions. Furthermore, prospective studies that include all factors that may influence the impact of dietary practices on diabetic patients are required. Moreover, collaborative research involving multiple regions of the country is suggested to provide a more balanced view of dietary practices and potential risk factors among diabetic patients.

## Data Availability

The dataset used and analyzed during the current study available from the corresponding author on reasonable request.

## Abbreviations

AOR: Adjusted odd ratio
CI: Confidence interval
COR: Crude odds ratio
CMHS: College of medicine and health sciences
DM: Diabetes mellitus
DSSQ: The diabetic social support questionnaire
FANTA: Stands for Food and Nutrition Technical Assistance
FBG: Fasting blood glucose
HFIAS: Household Food Insecurity Access Scale
NGO: Non-governmental Organization
SPSS: The Statistical Package for the Social Sciences
T2DM: Type 2 diabetes mellitus
WHO: World Health Organization
